# The impact of physical-mental mixed fatigue on landing biomechanics inter-joint coordination and injury risk in elite American football players

**DOI:** 10.3389/fpsyg.2026.1778145

**Published:** 2026-06-24

**Authors:** Zilong Wang, Huizi Cui, Jie Lu, Lingyue Meng, Bojie Xuan, Yuanwu Zhu, Pengfei Wang, Junzhu An, Qiuxia Zhang, Xiangdong Wang

**Affiliations:** 1 School of Physical Education, Jimei University, Xiamen, China; 2 School of Physical Education, Soochow University, Suzhou, China; 3 School of Physical Education, Ningbo University, Ningbo, China; 4 College of Physical Education and Sports, Beijing Normal University, Beijing, China; 5 Department of Sports and Leisure, Dongshin University, Naju, Jeollanam-do, Republic of Korea

**Keywords:** American football players, cognitive load, fatigue, injury risk, joint coordination, landing

## Abstract

**Objective:**

This study aimed to investigate the effects of physical-mental mixed fatigue (PMF) on lower limb Biomechanics, Inter-joint Coordination and potential injury risk during landing task in elite American football players.

**Methods:**

Twelve male collegiate American football players were recruited. A randomized crossover controlled design was employed, wherein participants randomly completed baseline testing, physical fatigue (PF) intervention, and PMF intervention. PMF was induced utilizing a cycle ergometer protocol concurrently performed with a Stroop cognitive task. Kinematic and kinetic data during a landing task were synchronously collected using a Vicon motion capture system and force plates. Statistical comparisons were performed using repeated-measures analysis of variance.

**Results:**

PMF specifically altered ankle joint kinematic patterns, manifested as a decreased ankle dorsiflexion angle (*p* < 0.05) and an increased inversion angle (*p* < 0.05) at the instant of peak vertical ground reaction force (vGRF), alongside an increased peak inversion angle throughout the landing cycle (*p* < 0.05). However, neither PMF nor PF significantly affected hip or knee kinematics, joint range of motion (RoM), joint contribution strategy, lower limb coordination indices, or landing impact load indicators (*p* > 0.05).

**Conclusion:**

The findings suggest that PMF specifically disrupts ankle biomechanics, potentially predisposing the joint to a posture that may be associated with higher injury risk. Nevertheless, highly trained athletes may utilize adaptive compensatory strategies developed through long-term training to maintain overall landing stability and impact load levels, thereby potentially offsetting the negative effects of PMF on neuromuscular control. When assessing injury risk associated with PMF, joint-level kinematic changes should be emphasized in addition to global indicators.

## 1 Introduction

American football is a team sport characterized by high-intensity physical collisions, complex tactical execution, and rapid decision-making (Alonso-Aubin et al., 2021). Players are required not only to perform frequent sprints, changes of direction, jumps, and tackles but also to continuously engage in tactical recognition, reactive judgment, and decision execution under high-pressure environments (Davis et al., 2021; Kasahara et al., 2008). This dual demand on physical and cognitive resources often results in a state of high fatigue during the latter stages of competition, yet athletes must maintain high levels of performance and tactical execution (Wang et al., 2025a). Consequently, a deeper understanding of the impact of fatigue, particularly mixed fatigue, on athletic performance and injury risk is crucial for optimizing training design, enhancing competitive performance, and preventing sports injuries.

The landing action is a common technical component in American football, such as stabilizing after catching a ball or landing from an abrupt stop during a break, the quality of which directly relates to performance and injury occurrence (Belcher et al., 2024; Meng et al., 2022). During landing, coordinated movement of the lower limb joints and rational distribution of impact loads are key to maintaining stability and preventing non-contact injuries (Ren et al., 2022b). Thus, landing tasks are often used as an important biomechanical model for assessing lower limb biomechanics and injury risk (Wang et al., 2024a).

Fatigue is a critical factor influencing athletic performance and on-field decision-making (Freire et al., 2021; Ren et al., 2022a). Physical fatigue (PF) refers to a state of declined physiological function following sustained intense exercise (Gan et al., 2025; Niu et al., 2025), whereas physical-mental mixed fatigue (PMF) is a composite fatigue state induced concurrently by physical and cognitive tasks (Wu et al., 2023; Zhang et al., 2015). PMF involves not only diminished function of the peripheral muscular system but also, more critically, impairs neuromuscular control efficacy through central mechanisms. Specifically, simultaneous physical and cognitive tasks compete for limited central processing resources, leading to abnormal activation in brain regions such as the prefrontal cortex and anterior cingulate cortex. This subsequently weakens the generation and descending conduction of motor commands and interferes with sensory-motor integration processes (Tanaka et al., 2014). Such central interference can reduce the excitability of the motor neuron pool, impair the precision and reactivity of multi-joint coordinated control, and thereby increase the risk of movement errors and injuries (Mehta and Agnew, 2012). Recent studies have indicated that the cognitive load induced by MF can impair movement performance (Wang et al., 2025b) and potentially elevate the risk of landing-related injuries in American football players (Wang et al., 2026). American football players frequently face both high physical loads and complex cognitive tasks during matches, a dual load that often induces PMF and may consequently exert more complex effects on neuromuscular control and movement coordination (Wang et al., 2025a). Under PMF, athletes’ neuromuscular control and sensory-motor integration capabilities may be compromised, potentially affecting the coordination and load distribution patterns of the lower limb joints during landing, thereby increasing injury risk. However, although existing studies have explored the impact of PF or MF alone on performance, less attention has been paid to the potential role of PMF on lower limb biomechanical coordination and injury risk in realistic competition scenarios. Current research has neither revealed the differential response patterns of lower limb joint kinematics and kinetics in American football players performing landing tasks under PMF, nor has it thoroughly explored changes in inter-joint coordination. Therefore, systematically investigating the effects of PMF on the motor control mechanisms of American football players holds significant theoretical and practical importance.

Given this, the present study aimed to investigate the effects of PMF on lower limb biomechanics, inter-joint coordination, and potential injury risk during landing in American football players. To specifically quantify “inter-joint coordination,” we analyzed the spatiotemporal coupling relationship between interacting joints (i.e., hip-knee and knee-ankle) using Continuous Relative Phase (CRP) metrics. Utilizing three-dimensional motion capture and force plate technology, we focused on analyzing the kinematic, kinetic, and inter-joint coordination characteristics of the dominant lower limb under PMF conditions to elucidate the interfering effects on motor control mechanisms. Specifically, we proposed three hypotheses: first, PMF would alter lower limb biomechanical characteristics during landing; second, PMF would lead to changes in inter-joint coordination indices, reflecting diminished neuromuscular control function; and third, PMF would increase landing impact loads and elevate injury risk indicators.

## 2 Methods

### 2.1 Participants

Using GPower 3.1.9 software, with power (1-β), Type I error (α), and effect size (f) set to 0.80, 0.05, and 0.4, respectively, based on previous studies (Wang et al., 2025a; Mathieu et al., 2022) and the planned statistical analysis, a minimum sample size of 12 was calculated. Ultimately, 12 male athletes were selected from the Soochow University American football team [age: 21.8 ± 1.3 years, height: 181.0 ± 3.5 cm, body mass: 80.0 ± 10.8 kg, training experience: 4.3 ± 1.2 years]. All participants were key players from a championship team in the Chinese University American Football League and were in a transitional period of recovery and adjustment during testing. The dominant leg was determined using the kick ball test (Ren et al., 2022b). All assessments and screenings were conducted by an experienced experimenter. Prior to the experiment, all participants were informed about the testing procedures and provided written informed consent. The study protocol received approval from the Soochow University Ethics Committee (Ethics Approval Number: SUDA20240626H03).

Participants were included if they had no history of lower limb joint or neuromuscular diseases or injuries within the past 6 months, possessed similar morphological characteristics, and maintained an optimal physical fitness level. Additionally, eligible participants were engaged in regular training during the study period. They were required to abstain from strenuous exercise and caffeine intake for 24 h prior to testing, avoid alcohol consumption for at least 1 week, and maintain a good psychological state without severe psychological disorders or significant mental stress.

### 2.2 Instrumentation

The primary experimental apparatus included a Vicon infrared three-dimensional (3D) motion capture system, Kistler 3D force plates, a cycle ergometer, and heart rate monitoring equipment. Kinematic data were acquired using a Vicon system equipped with eight infrared cameras (Model: MX13, Vicon Motion Systems, UK) and the Plug-in Gait Full Body model, with a sampling frequency set at 200 Hz. Kinetic data were collected synchronously using two Kistler 3D force plates (Model: 9281EA, Kistler Instrumente AG, Switzerland; dimensions: 90 cm × 60 cm × 10 cm) embedded in the center of the capture volume. The force plates operated at a sampling rate of 1,000 Hz and were synchronized with the motion capture system via an analog-to-digital converter. Additionally, the experimental intervention was conducted using a cycle ergometer (Model: POWERMAX-V II, Combi, Japan), while physiological load was continuously monitored throughout the sessions using a Bigrun Team chest-strap heart rate sensor.

### 2.3 Experimental design and procedures

**Figure 1:**
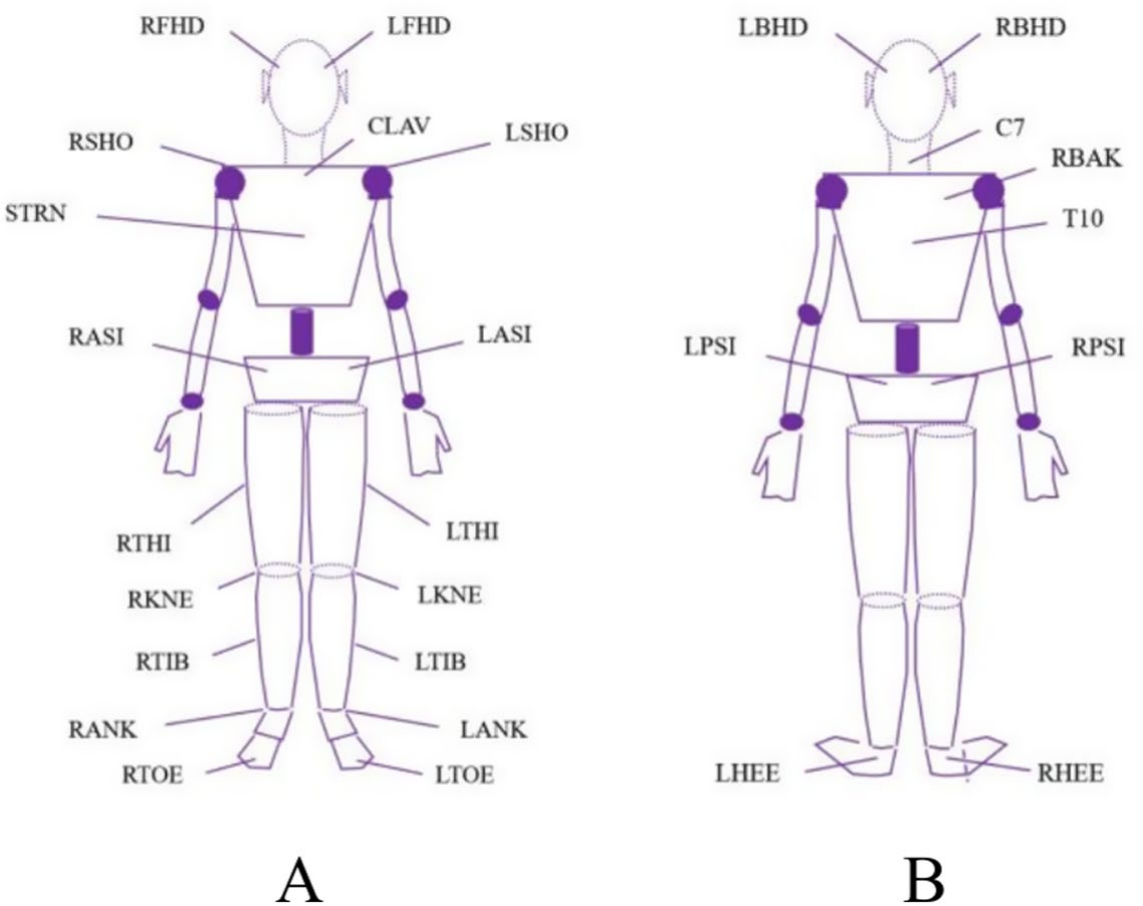
Marker point pasting. **(A)** Anterior view; **(B)** Posterior view. C7, Seventh cervical vertebra; CLAV, Clavicle midpoint; LANK: Left ankle; LBHD: Left back head; LFHD: Left front head; LSHO: Left acromion; LHEE: Left heel; LKNE: Left knee; LPSI: Left posterior superior iliac spine; LASI: Left anterior superior iliac spine; LTHI: Left thigh; LTOE: Left toe; LTIB: Left tibia; RANK: Right ankle; RASI: Right anterior superior iliac spine; RBHD: Right back head; RFHD: Right front head; RHEE: Right heel; RKNE: Right knee; RPSI: Right posterior superior iliac spine; RSHO: Right acromion; RTHI: Right thigh; RTIB: Right tibia; RTOE: Right toe; RBAK: Right scapula medial border; STRN: Sternum; T10: Tenth thoracic vertebra.

A randomized crossover trial design was employed. Prior to the formal experiment, all participants underwent a comprehensive familiarization session. This session ensured that participants fully mastered the Rating of Perceived Exertion (RPE, Borg 6–20 scale), the cycle ergometer protocol, the response rules for the Stroop cognitive task, and the execution of standard landing mechanics. This familiarization phase was implemented to minimize potential learning effects. The formal experiment consisted of three separate sessions attended sequentially, separated by a washout period of at least 1 week to prevent carryover effects. The first session served exclusively as the Baseline assessment, where participants performed the landing task without any prior fatigue intervention. The subsequent two sessions involved the PF and PMF interventions, the order of which was randomized (counterbalanced) across participants. In these intervention sessions, the corresponding landing task was conducted immediately after the cessation of the fatigue protocol (i.e., only post-fatigue measurements were recorded) to capture the acute effects of fatigue. Upon arrival for each session, participants changed into standardized tight-fitting shorts and athletic shoes provided by the laboratory. Following a standardized warm-up, an experienced technician attached 27 reflective markers (14 mm diameter) to specific anatomical landmarks in accordance with the full-body model protocol (Figure 1) to ensure consistency in marker placement. The corresponding movement test was conducted immediately after each intervention (PF or PMF). The testing time for each participant was kept consistent across all sessions, with deviations controlled within 2 h, to ensure that subjective fatigue perceptions were primarily attributable to the task load rather than circadian variations. The entire data collection was completed within 4 weeks.

### 2.4 Experimental protocol

#### 2.4.1 PF protocol

PF was induced using a cycle ergometer protocol (Wu et al., 2023). Participants began with 1 min of unloaded warm-up cycling, followed by a formal loading phase. The initial load was set at 50 W, increased by 50 W every 3 min until reaching 200 W, which was then maintained until the Participant reached the predetermined fatigue threshold. Fatigue level was monitored via heart rate and the Rating of Perceived Exertion (RPE) scale. Successful PF induction was defined as meeting either of the following criteria: (1) RPE reaching 17 or higher (“very hard”); or (2) Heart rate reaching 85% or more of the age-predicted maximum heart rate (220 - age) (Zhang et al., 2022).

#### 2.4.2 PMF protocol

The PMF protocol was designed with reference to Barzegarpoor et al. (2020) and de Lima-Junior et al. (2023), involving the concurrent performance of the Stroop cognitive task alongside the PF task. The specific procedure for the Stroop task was based on Wang et al. (2026). Four Chinese characters (“Red,” “Green,” “Blue,” “Yellow”) were presented sequentially in random order on a computer screen, displayed in one of the four corresponding colors. The probability of the character’s color not matching its meaning (incongruent stimuli) was set at 50%. Participants were instructed to verbally state the color of the character as quickly and accurately as possible. Each character was displayed for 1,000 ms, followed by a 1,000 ms blank screen interval before the next stimulus. The task was managed using E-prime 3.0 software. Two staff members supervised the process to ensure compliance. If a response was incorrect or no response was made within 1,500 ms, the staff manually triggered a system beep to alert and motivate the participant to maintain focus. The monitoring indicators for PMF induction (heart rate and RPE) were identical to those for PF.

#### 2.4.3 Landing task protocol

**Figure 2:**
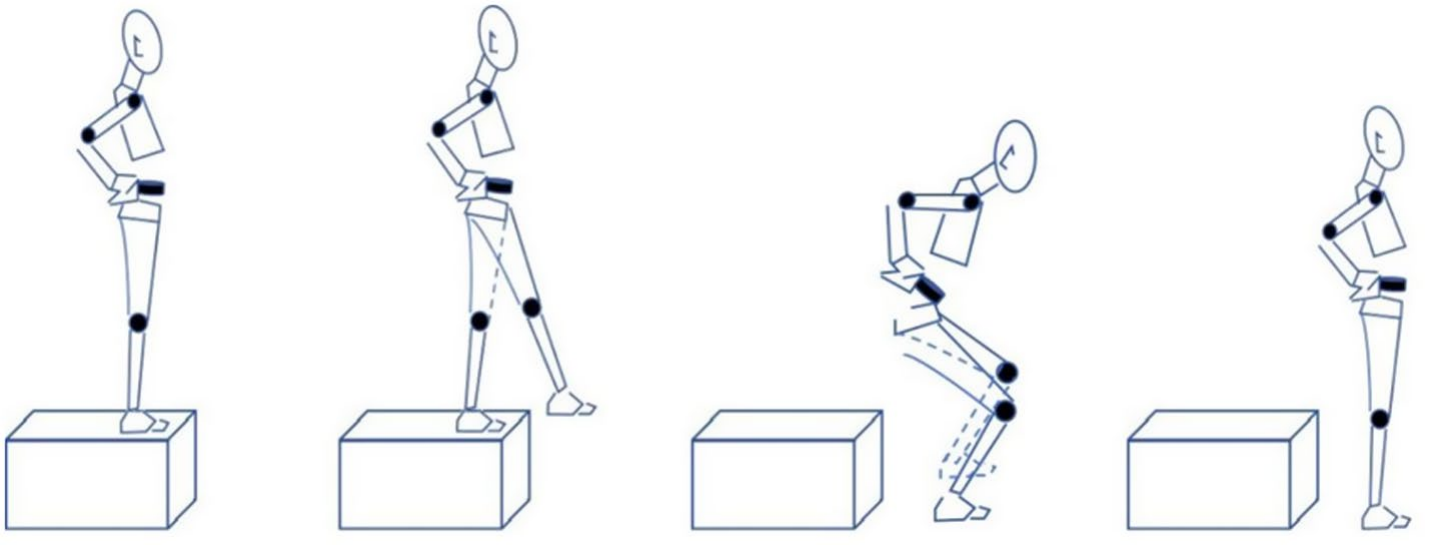
Schematic diagram of the landing action.

The landing task protocol was adapted from Ren et al. (2022a). Participants stood naturally on a 40 cm high plyometric box with their hands placed on their hips to minimize inertial effects from arm swing. Upon the “start” signal, participants stepped off the box with their dominant leg and dropped vertically without initial upward velocity, performing a double-leg landing using a “toe-heel” strategy (Figure 2). Sufficient practice was provided prior to formal testing to ensure participants were fully familiar with the landing technique. Three successful trials were recorded for each participant, separated by a 30-s rest interval to prevent fatigue accumulation. The mean values from the three trials were calculated for subsequent analysis. Due to the comprehensive familiarization session, participants demonstrated high proficiency, and data collection was typically completed within four attempts.

### 2.5 Data processing and analysis

Visual3D software was used to process the raw data collected by the Vicon system for kinematic and inverse dynamics analysis. A fourth-order low-pass Butterworth filter was applied to smooth the 3D coordinate data (cut-off frequency: 10 Hz) and force plate data (cut-off frequency: 50 Hz). Lower limb joint angles were calculated based on the Euler angle method (Wang et al., 2024b). The landing phase was defined as the period from the initial contact (IC), identified when the vertical ground reaction force (vGRF) first exceeded 10 N, until the time of maximum knee flexion angle. This period was normalized to 0–100% (Ren et al., 2022c).

Given that the dominant limb often bears higher loads during landing and its biomechanics are closely associated with injury risk (e.g., ACL injury, ankle sprain) (Zhang et al., 2023; Ren et al., 2022a), the dominant limb was selected for analysis to more effectively assess potential injury risk mechanisms under fatigue. The observed indicators included:

Regarding kinematics, we analyzed hip, knee, and ankle joint angles in the sagittal and frontal planes at initial contact (IC) and at the time of peak vGRF, as well as their peak values during the landing phase. Additionally, joint range of motion (RoM) was calculated as the difference between the angle at IC and the peak angle during the buffering phase. RoM was selected as a critical indicator of the kinetic chain’s capacity to dampen impact forces through joint excursion; reduced RoM typically signifies a “stiff” landing strategy, which is correlated with elevated ground reaction forces and increased injury potential.

For kinetics, the variables included peak vGRF normalized to body weight (BW), time to peak vGRF (T_vGRF), and vertical loading rate (LR). LR was defined as the ratio of peak vGRF to T_vGRF during the landing phase (Equation 1). Lower limb stiffness (Kleg, unit: BW/m) was determined as the ratio of peak vGRF to the change in lower limb length (ΔL) (Equation 2). Here, ΔL was operationally defined as the vertical displacement of the hip joint center from the instant of initial contact to the lowest point of the center of mass (corresponding to maximum knee flexion). Furthermore, joint contribution strategies were assessed using the knee-hip flexion contribution ratio (peak knee flexion / peak hip flexion) and the knee-ankle flexion contribution ratio (peak knee flexion / peak ankle dorsiflexion). These ratios were analyzed to determine the relative reliance on proximal versus distal joints for load dissipation, providing insight into compensatory redistribution strategies under fatigue.


LR=Peak vGRF/T_vGRF (1)

$$K_{\mathrm{leg}}=\frac{\text{Peak vGRF}}{\mathrm{\Delta L}}$$ (2)

To assess inter-joint coordination, the CRP for joint pairs was calculated. From this, the mean absolute relative phase (MARP) and deviation phase (DP) were extracted to evaluate the precision and stability of joint synergistic control. Quantifying inter-joint coordination allows for the detection of subtle neuromuscular control deficits, such as the decoupling of joint movements, which serves as a more sensitive marker for injury risk than discrete kinematic variables alone. MARP reflects the average coupling relationship (in-phase/anti-phase) of a joint pair, while DP represents the variability of the CRP curve throughout the time series. This method was adopted from Ren et al. (2022b). First, joint angles and angular velocities were normalized using amplitude-frequency normalization (Equations 3, 4) to reduce variability due to differences in movement amplitude and frequency. The relative phase angle φ for each joint was then calculated based on the normalized data and standardized via a four-quadrant phase diagram. CRP was calculated as the difference between the relative phase angles of the distal and proximal joints (Equation 5). Finally, MARP was calculated as the mean of the absolute CRP values (Equation 6), and DP was calculated as the standard deviation of CRP over the entire time series (Equation 7).


$$\omega {\left(i\right)}_{\mathrm{norm}}=\frac{\omega_{i}}{\max[\max(\omega_{i}),\max(-\omega_{i})]}$$ (3)

$$\theta {\left(i\right)}_{\mathrm{norm}}=\frac{2[\theta_{i}-\min(\theta_{i})]}{\max(\theta_{i})-\min(\theta_{i})}$$ (4)

$$\mathrm{CRP}(i)=\varphi_{\text{distal}}(i)-\varphi_{\text{proximal}}(i)$$ (5)

$$\text{MARP}=\frac{1}{N}\sum_{i=1}^{N}|\mathrm{CRP}(i)|$$ (6)

$$\mathrm{DP}=\sqrt{\frac{1}{N}\sum_{i=1}^{N}{\left[\mathrm{CRP}(i)-\bar{\mathrm{CRP}}\right]}^{2}}$$ (7)

### 2.6 Statistical analysis

Statistical analyses were performed using SPSS version 27.0. The normality of data distribution was assessed using the Shapiro–Wilk test, and homogeneity of variances was evaluated using Levene’s test. For normally distributed data, descriptive statistics are presented as mean ± standard deviation (Mean ± SD). A one-way repeated measures analysis of variance (ANOVA) was employed for statistical comparisons. Mauchly’s test was used to assess the assumption of sphericity; if this assumption was violated, the Greenhouse–Geisser correction was applied to adjust the degrees of freedom and *F*-values. Pairwise comparisons were conducted using the Bonferroni method. For non-normally distributed data, descriptive statistics are expressed as median and interquartile range [Mdn (IQR)]. Differences were analyzed using the Friedman test. Significant results were followed by *post hoc* comparisons using the Wilcoxon signed-rank test with a Bonferroni correction. All statistical tests were two-tailed, and the significance level was set at α = 0.05.

## 3 Results

### 3.1 Fatigue intervention

The average duration of the fatigue protocols was 24.5 ± 3.2 min for the PF condition and 24.3 ± 3.6 min for the PMF condition, with no statistically significant difference observed between conditions (*p* > 0.05). In both protocols, participants’ heart rates reached above 85% of their predicted maximum heart rates, and their subjective RPE scores reached 17 or higher. Thus, the participants met the prescribed criteria for successful fatigue induction.

### 3.2 Hip, knee, and ankle joint angles at IC and peak vGRF

**Figure 3:**
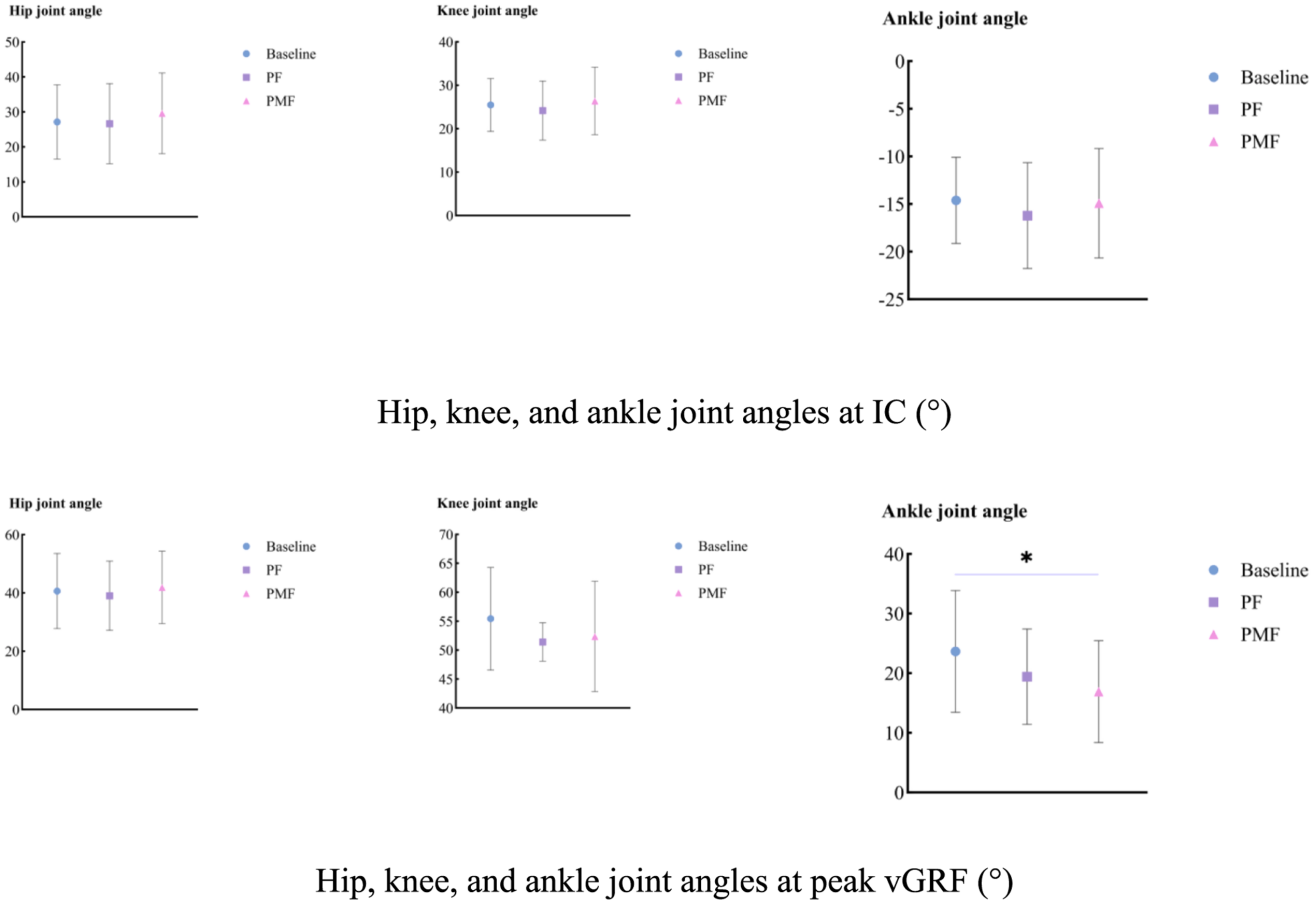
Sagittal plane hip, knee, and ankle joint angles at IC and at the time of peak vGRF during landing. Data are presented as Mean ± SD, *p < 0.05, The same applies below.

A significant main effect of fatigue condition was found for the ankle dorsiflexion angle at the time of peak vGRF (*p* = 0.003, *F* = 7.393, Eta^2^ = 0.402). Bonferroni *post-hoc* tests revealed that PMF resulted in a significantly smaller ankle dorsiflexion angle compared to Baseline (*p* = 0.011) (Figure 3).

**Figure 4:**
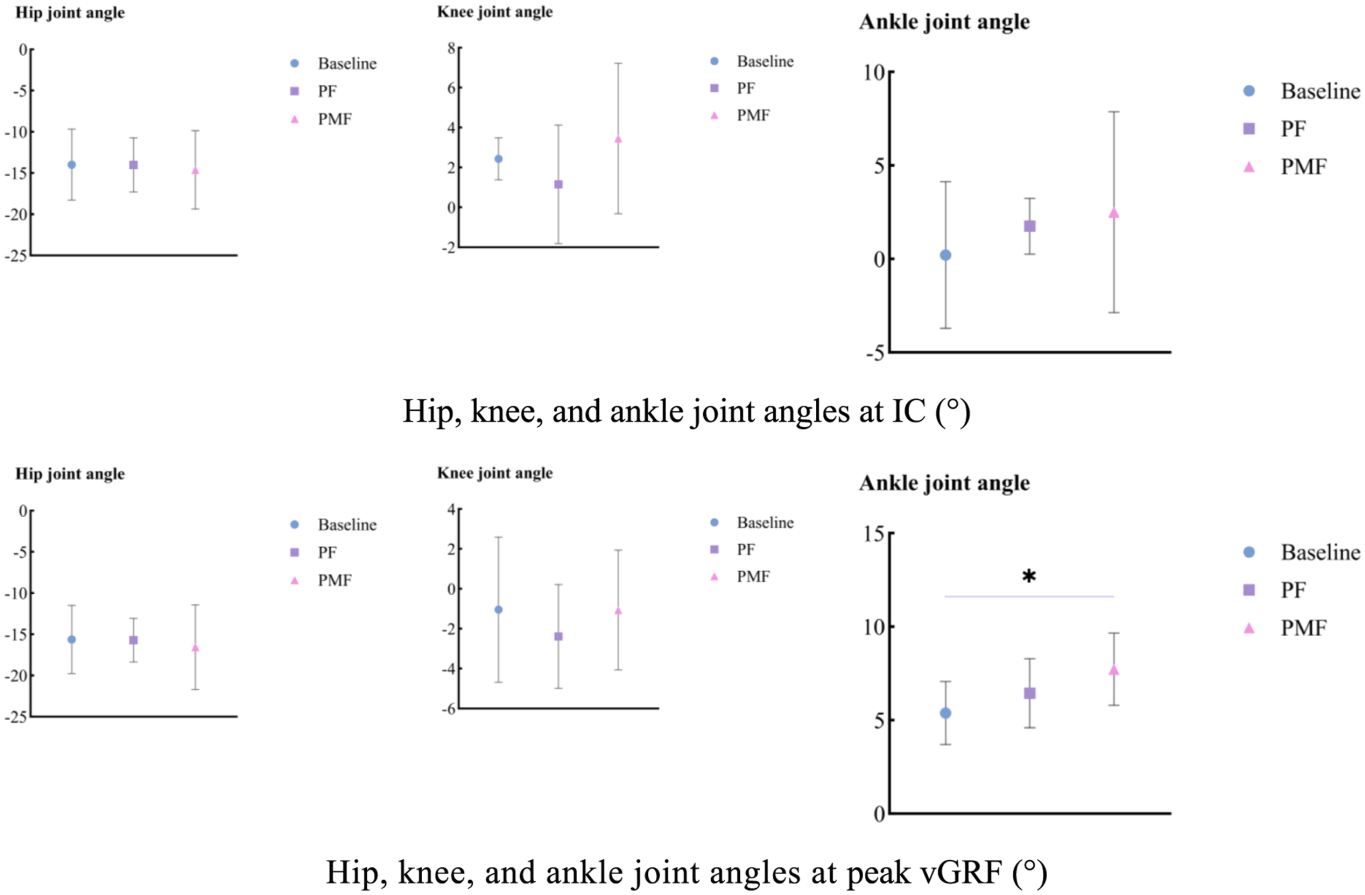
Frontal plane hip, knee, and ankle joint angles at IC and at the time of peak vGRF during landing.

A significant main effect of fatigue condition was found for the ankle inversion angle at the time of peak vGRF (*p* = 0.004, *F* = 7.385, Eta^2^ = 0.402). Bonferroni *post-hoc* tests revealed that PMF resulted in a significantly larger ankle inversion angle compared to Baseline (*p* = 0.004) (Figure 4).

### 3.3 Peak hip, knee, and ankle joint angles

**Figure 5:**
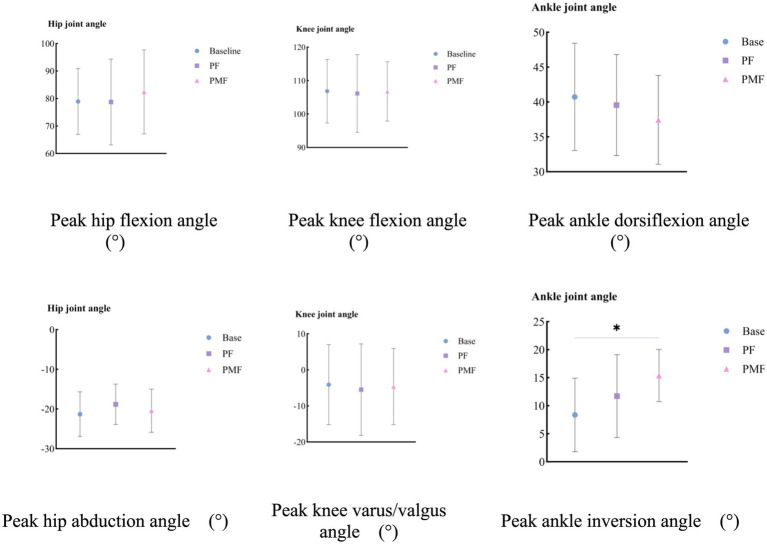
Peak angles at hip, knee, and ankle joints after landing.

A significant main effect of fatigue condition was found for the peak ankle inversion angle (*p* = 0.005, *F* = 6.834, Eta^2^ = 0.383). Bonferroni *post-hoc* tests revealed that PMF resulted in a significantly larger peak ankle inversion angle compared to Baseline (*p* = 0.012) (Figure 5).

### 3.4 Hip, knee, and ankle joint RoM

No statistically significant differences were observed in hip, knee, or ankle joint RoM indicators across the different fatigue conditions (*p* > 0.05) (Table 1).

**Table 1:** Hip, knee, and ankle RoM during landing.

Indicator	Baseline	PF	PMF	Statistical parameters
Hip flexion/extension RoM (°)	49.80 ± 6.51	50.36 ± 7.32	51.15 ± 11.53	*p* = 0.835
				*F* = 0.075
				Eta^2^ = 0.007
Knee flexion/extension RoM (°)	76.59 ± 8.93	77.44 ± 9.53	75.73 ± 7.25	*p* = 0.793
				*F* = 0.235
				Eta^2^ = 0.021
Ankle dorsiflexion/plantarflexion RoM (°)	48.43 ± 10.57	47.98 ± 9.75	45.30 ± 8.46	*p* = 0.242
				*F* = 1.515
				Eta^2^ = 0.121

### 3.5 Hip, knee, and ankle joint contribution strategy

No statistically significant differences were observed in the joint contribution strategy indicators across the different fatigue conditions (*p* > 0.05) (Table 2).

**Table 2:** Hip, knee, and ankle joint contribution strategies during landing.

Indicator	Baseline	PF	PMF	Statistical parameters
Knee-hip flexion ratio	1.38 ± 0.22	1.40 ± 0.31	1.34 ± 0.26	*p* = 0.600
				*F* = 0.315
				Eta^2^ = 0.028
Knee-ankle flexion ratio	2.72 ± 0.40	2.79 ± 0.73	2.93 ± 0.59	*p* = 0.367
				*F* = 0.951
				Eta^2^ = 0.080

### 3.6 Differences in relative phase indicators post-landing

No statistically significant differences were observed in the knee-hip or ankle-knee relative phase indicators (MARP, DP) across the different fatigue conditions (*p* > 0.05) (Table 3).

**Table 3:** Knee-hip and ankle-knee relative phase indicators during landing.

Indicator	Baseline	PF	PMF	Statistical parameters
MARP_Knee-Hip	4.19 ± 1.79	3.69 ± 1.46	3.90 ± 1.24	*p* = 0.686
				*F* = 0.383
				Eta^2^ = 0.034
DP_Knee-Hip	3.47 (3.22)	3.20 (1.41)	3.28 (2.16)	*p* = 0.558
				χ^2^ = 1.167
				Df = 2
MARP_Ankle-Knee	16.39 (3.10)	14.95 (5.71)	15.30 (2.91)	*p* = 0.368
				χ^2^ = 2.000
				Df = 2
DP_Ankle-Knee	13.11 (3.70)	13.11 (4.60)	12.44 (2.15)	*p* = 0.205
				χ^2^ = 3.167
				Df = 2

Due to the distribution characteristics of the data: (a) MARP_Knee-Hip follows a normal distribution and is presented as Mean ± Standard Deviation (M ± SD). (b) DP_Knee-Hip, MARP_Ankle-Knee, and DP_Ankle-Knee do not follow a normal distribution and are presented as Median (Interquartile Range) [Mdn (IQR)]. Statistical analysis was performed using the Friedman test.

### 3.7 Kinetic indicators

A significant main effect of fatigue condition was found for T_vGRF (*p* = 0.029, *F* = 4.189, Eta2 = 0.276). However, Bonferroni *post-hoc* comparisons did not reveal any statistically significant pairwise differences (*p* > 0.0167). No other kinetic indicators showed significant main effects (*p* > 0.05) (Table 4).

**Table 4:** Kinetic indicators during landing.

Indicator	Baseline	PF	PMF	Statistical parameters
Peak vGRF (BW)	1.80 ± 0.38	1.94 ± 0.43	2.00 ± 0.48	*p* = 0.509
				*F* = 0.696
				Eta^2^ = 0.060
T_vGRF (ms)	62.50 ± 20.90	54.20 ± 12.40	51.70 ± 17.00	***p* = 0.029**
				*F* = 4.189
				Eta^2^ = 0.276
LR (BW/ms)	0.34 ± 0.20	0.38 ± 0.13	0.43 ± 0.20	*p* = 0.136
				*F* = 2.191
				Eta^2^ = 0.166
K_leg_ (BW/m)	4.14 ± 0.98	4.61 ± 1.18	4.78 ± 1.40	*p* = 0.216
				*F* = 1.645
				Eta^2^ = 0.130

Bold value indicates p<0.05.

## 4 Discussion

### 4.1 Effects of PMF on lower limb biomechanical characteristics during landing

This study investigated the potential effects of PMF on the lower limb biomechanical characteristics of American football players during landing, aiming to elucidate the specific interference patterns of composite fatigue on motor control mechanisms and their practical implications for injury prevention and performance optimization. The results indicated that PMF affected ankle joint performance in both the sagittal and frontal planes, specifically manifesting as decreased ankle dorsiflexion and increased inversion at the time of peak vGRF, alongside an increased peak inversion angle throughout the movement cycle. This finding partially supports the first hypothesis, suggesting that PMF alters lower limb biomechanics during landing and may exert a more pronounced disruptive effect on ankle biomechanics compared to PF alone.

From a biomechanical perspective, the ankle joint plays a critical role in force transmission, and the observed alterations in its movement patterns can directly impact overall stability (Lee et al., 2020; Taylor et al., 2019). The observed reduction in dorsiflexion and increase in inversion may reflect decreased pre-activation levels and reactive control capacity of the muscles surrounding the ankle complex (specifically the dorsiflexors and evertors) under PMF, resulting in a less stable and more injury-prone joint position during the landing buffering phase. This result is consistent with the findings of Small et al. (2021), who reported that cognitive load can interfere with sensorimotor integration and reduce dynamic balance control. Furthermore, Tanaka et al. (2014), using magnetoencephalography, demonstrated that cognitive tasks can inhibit activity in the right anterior cingulate cortex during physical activity. Although the cognitive task was not performed concurrently with the landing, the residual central fatigue induced by PMF may impair the subsequent precision of neuromuscular control, leading to the specific kinematic alterations observed at the ankle joint level.

Notably, despite significant changes at the ankle, no significant effects of PMF or PF were found on hip or knee kinematics, joint RoM, joint contribution strategies, or indicators such as peak vGRF, LR, and K_leg_. This discrepancy between altered local kinematics (ankle) and unchanged global kinetics (vGRF, K_leg_) suggests an integrated compensatory mechanism. Specifically, the lower limb kinetic chain likely redistributed loads to mask the distal deficits caused by PMF, thereby maintaining overall landing stability. While such compensatory mechanisms may aid short-term performance maintenance, they could potentially increase the risk of coordinative imbalances within the kinetic chain over the long term (Lee et al., 2018; Taylor et al., 2022; Kratzer et al., 2025). Furthermore, the lack of significant effects from PF alone on any indicators might be attributed to the high training level of the participants. While long-term training provides physical adaptability, we also acknowledge that the cycling protocol, lacking the eccentric loading and multi-directional demands of football, may not have induced sufficient fatigue to alter proximal joint mechanics. This finding resonates with the conclusions of Wang et al. (2025a,b), who also noted that professional American football players could maintain stability in certain biomechanical indicators under PF, highlighting their excellent physical reserve and neuromuscular adaptability. In contrast, PMF, by adding a cognitive task, might more readily expose potential deficits in neuromuscular control. This aligns with the results of Mehta and Agnew (2012), who found that additional cognitive load could exacerbate perceived muscle fatigue and reduce contraction efficiency. Studies by Barzegarpoor et al. (2020) and Díaz-García (2023), utilizing Stroop tasks combined with cycling, found that the combination of PF and cognitive load exacerbated declines in athletic performance, further corroborating the negative impact of PMF on motor control. Notably, Wang et al. (2025a,b) also found more pronounced effects of PMF on indicators such as the reactive strength index and ratio, suggesting that PMF might further exacerbate ankle instability during landing by affecting rapid muscle reaction capacity. This, together with the observed increase in inversion and decrease in dorsiflexion in the present study, points toward a comprehensive interfering effect of PMF on lower limb motor control.

In summary, PMF likely specifically affects ankle biomechanics through central-peripheral interactions without altering global kinetic indicators, suggesting its impact is joint-specific and strategically compensated. Therefore, changes in ankle biomechanical patterns in athletes during the latter stages of competition or under high cognitive-physical dual-task conditions should be thoroughly considered and targeted for injury risk monitoring and intervention.

### 4.2 Effects of PMF on lower limb coordinative control

The second hypothesis postulated that PMF would lead to changes in lower limb inter-joint coordination indices, reflecting diminished neuromuscular control function. However, the study found no significant effects of PMF or PF on MARP and DP, nor any changes in joint contribution strategies. This result did not support the second hypothesis.

From the perspective of neural control and movement inter-joint coordination, synergistic movement of multiple lower limb joints relies on precise regulation of multiple muscle groups by the central nervous system and efficient sensory-motor integration (Asadi and Erfanian, 2012; Hoogland et al., 2015). Although PMF might interfere with attention allocation and motor planning through the combined effects of PF and cognitive load, highly trained athletes often exhibit stronger movement automaticity and adaptive control capabilities (Fan et al., 2025; Riscart-López et al., 2025), allowing them to maintain basic inter-joint coordination patterns even under mixed fatigue states. This “neuromuscular redundancy” is likely an adaptive phenomenon induced by long-term specialized training (Avela et al., 2006; Dayan and Cohen, 2011), involving mechanisms such as optimized motor unit recruitment by the central system, enhanced reflex regulation at the spinal level, and improved sensory feedback efficiency (Zemková and Zapletalová, 2022; Strniště et al., 2019). Consequently, even under general fatigue, athletes might partially compensate for losses in control precision through pre-activation mechanisms and rapid reflex pathways, thereby maintaining basic stability in inter-joint coordination patterns. This view aligns with the conclusions of Morita et al. (2022, 2023) from studies on wheelchair racing athletes, indicating that long-term training can enhance motor cortex function and improve inter-joint coordination and adaptation abilities. Similarly, Prieske et al. (2017) noted that although fatigue impairs motor control, elite athletes can effectively reduce variability in relevant biomechanical indicators by increasing joint stiffness and optimizing muscle co-activation strategies. This phenomenon reflects their advantage in neuromuscular control mechanisms, allowing them to maintain relative stability in inter-joint coordination through efficient compensation under fatigue interference. Additionally, the landing task used in this study is common in American football competition and training, and all participants were given ample practice opportunities before formal testing, likely resulting in highly skilled movement patterns. This might mean execution relies more on basic control mechanisms at the spinal and subcortical levels rather than on movement adjustments requiring high-level cognitive involvement. Therefore, although the PMF state might increase central processing load, it may not be sufficient to disrupt the highly automated landing inter-joint coordination pattern. This speculation is partially consistent with the recent findings of Baiguzhin and Sashenkov (2025) in university athletes, which showed that non-athletes often exhibited longer inhibition reaction times and increased sympathetic nervous activity under cognitive load, indicating their nervous systems required more resources to maintain stability; whereas athletes maintained stable performance in cognitive tests and showed higher parasympathetic activity, reflecting higher neural efficiency in responding to cognitive demands. Furthermore, inter-joint coordination indices might be less sensitive to fatigue than single-joint kinematic indicators, potentially requiring longer duration or higher intensity interventions to show significant changes. Future studies could introduce paradigms with higher cognitive-motor dual-task loads or more complex movement decisions, such as side-step landings or cutting maneuvers, to overcome the potential “ceiling effect” of the current task and more sensitively reveal the deeper effects of PMF on lower limb coordinative control.

It is noteworthy that although overall inter-joint coordination indices did not change significantly, the potential impact of PMF on neuromuscular control quality cannot be entirely ruled out. This apparent “stability” might not stem from undisturbed neural control but rather result from athletes actively maintaining performance through a series of compensatory strategies. Specifically, leveraging adaptive advantages formed through long-term training, athletes under PMF might enhance muscle co-activation levels (e.g., co-contraction of agonist and antagonist muscles to increase joint stability) and adjust intramuscular inter-joint coordination strategies to optimize motor unit recruitment order and joint strategies (Granacher et al., 2016), thereby compensating for decreased output due to local muscle fatigue. These strategies might “mask” potential coordinative variability, keeping overall inter-joint coordination indicators within a relatively stable range. This is also consistent with the discussion above regarding ankle kinematic changes. These adaptive adjustments at specific joint levels can be viewed as compensatory mechanisms employed locally by the neuromuscular system to cope with fatigue, aiming to maintain the overall quality of task execution, particularly by preventing degradation of the inter-joint coordination pattern. Future research could incorporate electromyographic (EMG) coherence analysis to investigate changes in corticomuscular functional coupling pre- and post-fatigue. Complexity metrics quantifying nonlinear features of surface EMG signals or movement time-series data could also be employed to more sensitively capture subtle alterations in neural control strategies under fatigue. These methods hold promise for revealing, at a more fundamental level, how PMF affects the precise neural regulation of movement and how athletes compensate to maintain performance.

In conclusion, although PMF did not induce significant changes in inter-joint coordination indices, this does not necessarily indicate an absence of effect on neuromuscular control function. Future studies might consider employing more complex task paradigms, more sensitive analytical methods, or more extreme fatigue induction protocols to reveal the potential impact of PMF on coordinative control. Simultaneously, multimodal data (e.g., EMG, EEG) should be integrated to comprehensively assess the effects of PMF on neuromuscular inter-joint coordination across different levels.

### 4.3 Potential impact of PMF on landing impact load and injury risk

The third hypothesis proposed that landing impact load would significantly increase under PMF, leading to elevated injury risk indicators. However, the results showed no significant changes in peak vGRF, T_vGRF, LR, or Kleg, which is inconsistent with the third hypothesis. Although statistical significance was not observed for most indicators, as Abadie (2020) stated, “results consistent with the null hypothesis are equally foundational for theoretical revision, and non-significant results should be explicitly reported and discussed.”

From a biomechanical and injury mechanism perspective, the magnitude of landing impact load is influenced not only by fatigue state but also by landing strategy, joint stiffness regulation, and energy absorption mechanisms. In this study, despite the trend toward increased inversion and decreased dorsiflexion at the ankle, the athletes might have dispersed the impact force to some extent by adjusting overall posture or muscle co-activation strategies, preventing an increase in global load indicators. This compensatory adjustment could be an adaptive response employed by athletes when facing diminished neuromuscular control capacity, aimed at maintaining overall landing stability (Ren et al., 2022a). Furthermore, professional athletes likely develop optimized landing patterns through long-term training, enabling them to maintain relatively stable mechanical output via compensatory mechanisms even under fatigue, consistent with the discussion above.

It is noteworthy that increased ankle inversion angle is typically closely associated with a higher risk of ankle sprains, while reduced dorsiflexion may increase anterior translational force and Achilles tendon loading (Purevsuren et al., 2018; Zhang et al., 2025). Even if global kinetic indicators remain unchanged, the redistribution of local joint loads could potentially elevate injury risk, particularly for athletes under high pressure during the latter stages of competition. This result suggests that when assessing the impact of fatigue on injury risk, joint-level kinematic changes and local load characteristics should be emphasized alongside global mechanical indicators. Previous studies have indicated that abnormal local joint mechanics can be a potential mechanism for lower limb non-contact injuries, potentially increasing risk even in the absence of changes in global load indicators (Wannop and Stefanyshyn, 2016).

Additionally, the lack of observed effect of PF or PMF on Kleg might be related to athletes adjusting muscle stiffness to maintain overall mechanical properties. Lower limb stiffness, resulting from the combined action of active muscle regulation and passive tissue properties, its stability under fatigue might reflect overall optimization of motor behavior by the central system through mechanisms such as optimizing motor unit recruitment, coordinating muscle co-activation, and adjusting tendon elasticity (Padua et al., 2006; Xu et al., 2025). Professional athletes, after long-term specialized training, likely develop adaptive strategies that allow them to effectively maintain lower limb stiffness even under fatigued conditions. For instance, as discussed, they might compensate for fatigue-induced declines in mechanical performance by altering lower limb joint movement patterns and the activation sequence of surrounding muscle groups, as observed in the changes in ankle kinematics in this study. It is worth exploring further that this “stability” in global mechanical indicators might mask excessive loading on local tissues, thereby increasing the risk of cumulative injuries under long-term or repeated exposure.

From an injury prevention perspective, the results of this study emphasize the necessity for PMF-specific monitoring and intervention in athletes. Traditional injury risk assessment methods based solely on global mechanical indicators may fail to effectively capture the subtle yet important biomechanical alterations induced by PMF. Therefore, future efforts should consider developing comprehensive, multi-modal assessment protocols that integrate EMG activity, musculoskeletal modeling computations, and real-time cognitive-motor dual-task load monitoring on the foundation of traditional kinetic and kinematic analysis. This would allow for a holistic identification of high-risk states from multiple dimensions: neural control, mechanical load, and cognitive resource allocation. Such integrated strategies would facilitate earlier detection of potential injury signals and provide a theoretical basis for developing targeted fatigue management and injury prevention interventions.

In summary, although PMF did not cause a significant increase in landing impact load, by altering ankle movement patterns, it may still pose a potential threat to athletes’ injury risk. Future research could combine EMG analysis, finite element modeling, and long-term tracking methods to further elucidate the relationship between local load changes and injury mechanisms, providing a scientific basis for formulating targeted prevention strategies.

### 4.4 Limitations

This study has several limitations that must be acknowledged. First, the participants were all high-level male collegiate American football players from a single championship team, resulting in a relatively limited sample size and the absence of female athletes, which restricts the generalizability of the findings to other populations. Second, although standardized fatigue protocols and landing tasks were used, the laboratory environment differs from real-game scenarios, and cognitive task performance was not monitored, potentially limiting the ecological validity. Third, the study primarily relied on kinematic and kinetic indicators to assess lower limb biomechanics, lacking direct measurements of neuromuscular control mechanisms; therefore, discussions regarding compensatory strategies remain somewhat speculative without direct physiological evidence. Fourth, it is acknowledged that the study compared post-fatigue performance (from intervention sessions) to a separate baseline session, rather than conducting pre- and post-fatigue trials within the same visit. While this design minimizes intra-session fatigue accumulation and testing duration, it does not account for potential day-to-day biological variability in landing biomechanics. Finally, subjective mental fatigue was not assessed using a specific scale (e.g., VAS or NASA-TLX); instead, we relied on RPE and Heart Rate to monitor overall exertion, which does not specifically isolate the cognitive component of fatigue from physical exertion. To enhance generalizability, future studies should aim to expand the sample size, include female athletes for sex-based comparisons, and involve athletes from diverse competitive levels or sports disciplines. To improve ecological validity, researchers should incorporate more realistic game-like scenarios (e.g., virtual reality or field-based protocols) and explicitly monitor cognitive performance metrics (e.g., reaction time and accuracy) to better simulate real-match demands. Furthermore, integrating electromyography (EMG) or electroencephalography (EEG) would provide direct physiological evidence of neuromuscular control adaptations and corticomuscular coupling under fatigue. Future experimental designs could also employ pre- and post-testing within the same session or utilize longitudinal tracking methods to better control for day-to-day biological variations. Lastly, future investigations should utilize specific validated scales (e.g., NASA-TLX or VAS for mental fatigue) alongside physiological measures to more precisely isolate and quantify the cognitive dimension of fatigue.

## 5 Conclusion

This study investigated the potential effects of PMF on lower limb biomechanical inter-joint coordination and injury risk during landing in elite American football players. The findings indicate that PMF specifically alters ankle joint kinematic patterns, manifested as decreased dorsiflexion and increased inversion angles during the landing buffering phase, suggesting the ankle assumes a biomechanically less stable and potentially more injury-prone position. However, PMF did not lead to changes in hip or knee kinematics, overall inter-joint coordination indices, or landing impact loads. This suggests that highly trained athletes may utilize adaptive compensatory strategies developed through long-term training to maintain overall landing stability, potentially “masking” the negative effects of PMF on neuromuscular control. Nonetheless, localized anomalies at the ankle may still potentially increase its sprain risk. The results emphasize that when assessing fatigue-related injury risk, joint-level kinematic changes should be prioritized alongside global kinetic loads. Furthermore, traditional biomechanical indicators might be insufficient to sensitively capture the full impact of PMF; future efforts should develop multi-modal assessment schemes integrating neuromuscular and cognitive monitoring to provide a theoretical basis and practical guidance for more precise fatigue monitoring and injury prevention.

## Data Availability

The original contributions presented in the study are included in the article/supplementary material, further inquiries can be directed to the corresponding authors.
